# Hair follicle stem cell fate supports distinct clinical endotypes in hidradenitis suppurativa

**DOI:** 10.1111/jdv.70152

**Published:** 2025-11-06

**Authors:** Audrey Onfroy, Francette Jean‐Louis, Philippe Le Corvoisier, Fanny Coulpier, Kévin Muret, Eric Bonnet, Raphaele Arrouasse, Caroline Boucle, Salwa Abid, Emilie Sbidian, Christina Bergqvist, Pierre Wolkenstein, Véronique Godot, Jean‐François Deleuze, Yves Lévy, Piotr Topilko, Etienne Audureau, Sophie Hue

**Affiliations:** ^1^ INSERM U955‐Team 9, Institut Mondor de Recherche Biomédicale (IMRB) Université Paris‐Est Créteil (UPEC) Créteil France; ^2^ Vaccine Research Institute (VRI) Créteil France; ^3^ INSERM, Clinical Investigation Center 1430, AP‐HP, DMU Saphire Henri Mondor University Hospital Créteil France; ^4^ Institut de Biologie François Jacob, CEA, Centre National de Recherche en Génomique Humaine (CNRGH) Université Paris‐Saclay Evry France; ^5^ EpiDermE EA 7379 Université Paris Est Créteil Créteil France; ^6^ Department of Dermatology Assistance Publique‐Hôpitaux de Paris, Henri Mondor Hospital Créteil France; ^7^ Public Health Department APHP, Hospital Henri‐Mondor Créteil France; ^8^ Biologic Immunology‐Hematology Department, DMU Biologie APHP, Hospital Henri‐Mondor Créteil France

**Keywords:** endotypes, hair follicle cells, hidradenitis suppurativa, inflammation

## Abstract

**Background:**

Hidradenitis suppurativa (HS) is a chronic inflammatory skin disease affecting approximately 1% of the global population. Its pathogenesis involves both aberrant keratinization and autoinflammation, but the temporal relationship between these processes remains unclear.

**Objectives:**

To investigate the role of HF stem cell (HF‐SC) fate in the early pathogenesis of HS.

**Methods:**

We performed single‐cell RNA sequencing of HF cell populations from HS patients and healthy donors. We then analysed HF cell composition isolated from perilesional skin of 49 HS patients and integrated these data with clinical phenotypes to define disease endotypes.

**Results:**

Our data revealed two distinct differentiation trajectories of HF stem cells (HF‐SCs): one leading to interfollicular epidermis (IFE) basal cells enriched in inflammatory pathways, and another giving rise to outer root sheath (ORS) cells associated with keratinization. In HS lesions, both populations displayed altered inflammatory phenotypes and were closely linked to immune cell infiltration, pointing to a role in disease heterogeneity. By integrating clinical features with HF cell composition from 49 HS patients, we identified three major endotypes: (i) an inflammatory subtype, marked by T cell infiltration and an expansion of IFE basal cells; (ii) a keratinizing subtype, characterized by ORS enrichment and minimal inflammation; and (iii) a mixed subtype, exhibiting features of follicular remodelling, fistula formation and variable immune involvement.

**Conclusions:**

These findings provide novel insights into the epithelial‐immune interactions that drive HS and support a stratified therapeutic approach tailored to distinct epithelial dysfunctions.

## Plain Language Summary

Hidradenitis suppurativa (HS) is a chronic and painful inflammatory skin disease that affects about 1% of the population worldwide. Its causes are not fully understood, and this lack of knowledge limits the development of effective treatments.

In this study, conducted in France, we investigated the role of hair follicle stem cells (HF‐SCs) in HS. Firstly, using single‐cell transcriptomics data, we compared hair follicle (HF) cell populations between HS patients and healthy donors. Secondly, we analysed the composition of HFs in skin biopsies from 49 patients and linked these data with their clinical features.

We found that HF‐SCs can differentiate into two HF populations: (i) the basal interfollicular epidermis, associated with inflammation and, (ii) the outer root sheath, associated with keratinization. In the context of HS, these populations further displayed an altered inflammatory phenotype and were associated with the infiltration of immune cells. Finally, the integrated analysis of clinical features and HF cell composition identified three main endotypes. Each endotype displays a specific enrichment in HF cells and is related to keratinization, inflammation or both.

These findings suggest that the identified HF cell populations and their markers could serve as novel diagnostic biomarkers and therapeutic targets for HS.

## Graphical abstract

Hair follicles (HFs) were extracted from skin biopsies. From these, original sequencing data were obtained and integrated with two independent studies to build a HF single‐cell transcriptomic atlas. It enabled delineating the fate of HF stem cells. Clustering analysis of a patient cohort identified three endotypes, advancing personalized medicine development.

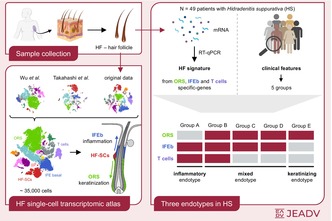

**Why was the study undertaken?**

- Hidradenitis suppurativa (HS) is a severe skin disorder involving aberrant keratinization and immune activation. It remains unclear whether autoinflammatory events precede or follow hyperkeratotic changes in hair follicle (HF) epithelia.

**What does this study add?**

- We generated the first single‐cell transcriptomic atlas of HF cells from HS patients.
- Cells from the middle outer root sheath (ORS) and from interfollicular epidermis (IFE) form two differentiation branches from HF stem cells, with distinct biological activities: keratinization and inflammation.
- In an independent cohort of 49 patients, we define three clinical endotypes, each associated with a specific epithelial trajectory and immune profile.

**What are the implications of this study for disease understanding and clinical care?**

- Perilesional skin in HS shows imbalanced HF‐SC differentiation that correlates with clinical features. The abundance of IFE and ORS cells may serve as novel biomarkers and therapeutic targets for personalized management of HS.

## INTRODUCTION

Hidradenitis suppurativa (HS) is a chronic, severe, inflammatory skin disorder with a prevalence of around 1% exerting a profound impact on quality of life.^1^ Characteristic lesions such as inflammatory nodules, abscesses and sinus tracts develop in axillary, inguinal and gluteal areas, typically around puberty. HS is a complex disease with contributory factors of genetic, epigenetic hormonal, mechanical, microbial and lifestyle factors such as obesity and smoking. Current therapeutic options remain limited, and treatment outcomes are often unsatisfactory, largely due to an incomplete understanding of HS pathogenesis.^2^ Moreover, pronounced disease heterogeneity is increasingly recognized as a key factor influencing clinical response.^3^ Distinct molecular and clinical endotypes may contribute to the failure of uniform therapies, highlighting the urgent need for personalized approaches.

HS is recognized as an autoinflammatory keratinization disease because the pathogenic mechanisms of HS are intimately associated with aberrant keratinization and autoinflammation.^4^ In unaffected skin from predilection sites, the infundibular outer root sheath (ORS) shows hyperplasia and hyperkeratosis likely driven by an intrinsic dysregulation of the hair follicle stem cell (HF‐SC) compartment. Notably, ORS cells exhibit replication stress, activating Type I interferon responses.^5^ These epithelial alterations, in conjunction with external factors such as obesity and smoking, contribute to follicle occlusion and the accumulation of keratin and bacteria within the dilated hair follicle. The subsequent formation and rupture of cysts induce acute inflammation, characterized by an immune infiltrate of neutrophils, macrophages, dendritic cells and T and B cells and increased expression of proinflammatory cytokines including IL1β, IL17 and TNFα.^6^ Treatment targeting inflammation, such as adalimumab, achieved clinical response in only 40%–60% of patients, and newer biological therapies have not significantly exceeded this response rate.^7^ A major barrier remains our limited understanding of how keratinization and inflammation interact.

In this study, we sought to better define the epithelial dynamics underpinning HS pathogenesis. Using single‐cell RNA sequencing (scRNA‐Seq), we characterized human HF cell populations and uncovered two distinct differentiation trajectories of HF‐SCs: one leading to interfollicular epidermal (IFE) basal cells enriched in inflammatory pathways, and the other to outer root sheath (ORS) cells, associated with keratinization. In HS patients, we observed that HF‐SCs, IFE basal cells and ORS cells exhibited a pro‐inflammatory transcriptional profile and were closely associated with immune cell infiltration. Based on these observations, we hypothesized that the trajectory of HF‐SC differentiation toward either lineage may influence disease heterogeneity. To investigate this, we analysed HF cell composition in a cohort of 49 HS patients and identified three major endotypes: (i) an inflammatory endotype, marked by T cell infiltration and expansion of IFE basal cells; (ii) a keratinizing endotype, enriched in ORS with minimal inflammation; and (iii) a mixed endotype, featuring follicular remodelling, fistula formation and variable levels of inflammation.

Our findings highlight the existence of distinct pathogenic mechanisms underlying HS—primarily inflammation, keratinization, or a combination of both—each rooted in specific HF‐SC trajectories. This mechanistic stratification offers a framework for understanding HS heterogeneity and supports personalized therapeutic strategies tailored to dominant epithelial dysfunctions.

## MATERIALS AND METHODS

Patients with hidradenitis suppurativa were recruited from the Fol‐HYDRA study and in accordance with ethics regulation. For scRNA‐Seq analysis, hair follicles were pulled out from skin samples from perilesional areas. After dedicated sample processing, scRNA‐Seq data were obtained following the 10X Genomics technology. Original count matrices and public scRNA‐Seq data (IDs OEP002321 and GSE129611) were analysed using the R language. Everything is provided to ensure the reproducibility of the analysis. For RT‐qPCR, the expressions of three genes specific for T cells, IFE basal cells and ORS cells as well as OAZ1 for reference, were measured from the RNA of pulled hair follicles. Whole genome sequencing data were obtained using Illumina technology and analysed through a dedicated pipeline. The clustering analysis was performed using a self‐organizing maps algorithm. Statistical analyses were made using appropriate tests.

Full descriptions of patient recruitment, sample collection, obtention of single‐cell RNA sequencing data and their analysis and the clustering analysis of clinical features of the FolHydra cohort are provided in Appendix S1.

## RESULTS

### Defining human hair follicle cell populations via integrated scRNA‐Seq analysis

**FIGURE 1:**
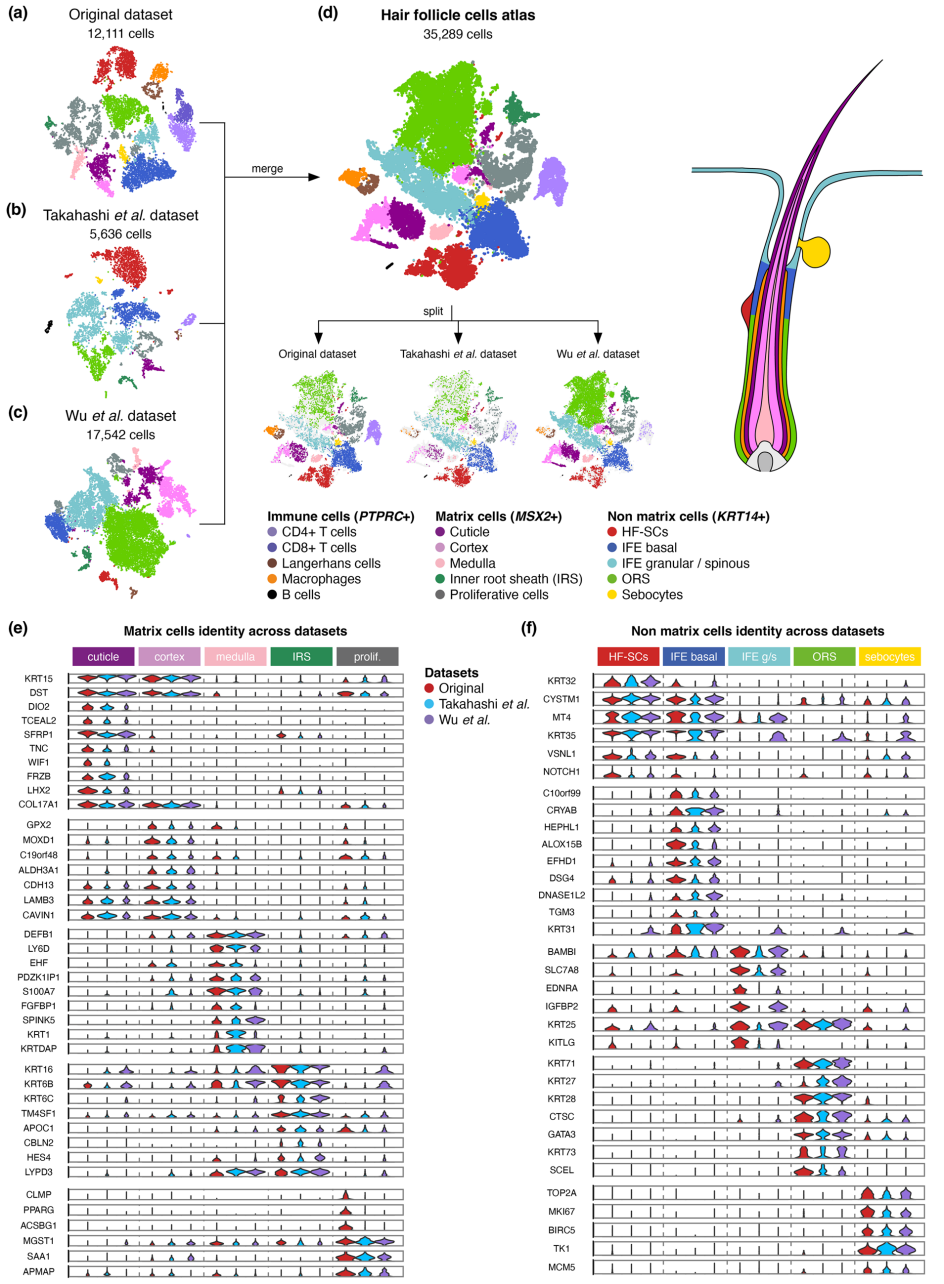
Transcriptomic atlas of hair follicle cells. (a–c) tSNE plots showing three individual datasets: Our dataset (a), Wu et al. dataset (b) and Takahashi et al. dataset (c), coloured according to cell type annotation. (d) After merging, the combined dataset contains 35,259 cells, coloured according to cell type annotation. Split view shows the overlapping for all cell types across datasets. (e, f) Violin plots within matrix (e) or non‐matrix (f) cells. Each panel is divided into five columns, assessing the identity of the cell type indicated on the top. Each column depicts three violin plots, associated with each dataset, showing the conserved expression of genes across dataset and laboratory. The legend key is shared between all panels.

Since hair follicles play a central role in HS pathology, we decided to perform scRNA‐Seq on HF cells. Skin samples were obtained from the perilesional lesions of five HS patients and compared to two healthy donors (HD) (see Table S1 for patients' information). A scRNA‐Seq analysis was conducted on HF cells, resulting in the acquisition of 12,111 cells that met the quality control criteria. The cells were annotated based on established HF markers (Table S2) and categorized into three main groups: immune cells, matrix cells and non‐matrix cells (Figure 1a). The immune cells included *CD4* ^+^ and *CD8* ^+^ T cells, Langerhans cells, macrophages and B cells.

To refine cell type annotation, we incorporated publicly available scRNA‐Seq data from healthy individuals^8^, ^9^ into a joint analysis with our dataset (Figure 1b,c). By merging these datasets, we constructed a comprehensive atlas of hair follicle (HF) cells (Figure 1d). This integrative approach enabled the identification of shared gene signatures across datasets, thereby enhancing the resolution and categorization of HF cell populations. The matrix category was further divided into five populations based on their transcriptional profiles: (i) cuticle (*KRT32*, *KRT35*), (ii) cortex (*KRT31*), (iii) medulla (*BAMBI*), (iv) inner root sheath (IRS) (*KRT71*, *KRT28*) and (v) proliferative cells, expressing high levels of *TOP2A* and *MKI67* genes (Figure 1e). The non‐matrix category also regroups five populations: (i) hair follicle stem cells (HF‐SCs) expressing high levels of *KRT15*, *COL17A1*, *DIO2* and *TCEAL2*; (ii) basal interfollicular epidermis (IFE) expressing *KRT15* and *COL17A1* but lacking *DIO2* and *TCEAL2* expression; (iii) granular and spinous IFE expressing *SPINK5* and *LY6D*; (iv) outer root sheath cells (ORS) expressing *KRT16, KRT6B* and *KRT6C*; and (v) sebocytes expressing *CLMP* and *PPARG* (Figure 1f).

This comprehensive molecular annotation revealed 15 transcriptionally distinct HF cell populations, consistently observed in both HS patients and healthy donors, irrespective of sex or sampling site (Figure S1). These findings provide a high‐resolution view of HF cellular diversity.

### HF‐SC fate mapping reveals two distinct lineages: ORS and IFE basal with unique molecular signatures

**FIGURE 2:**
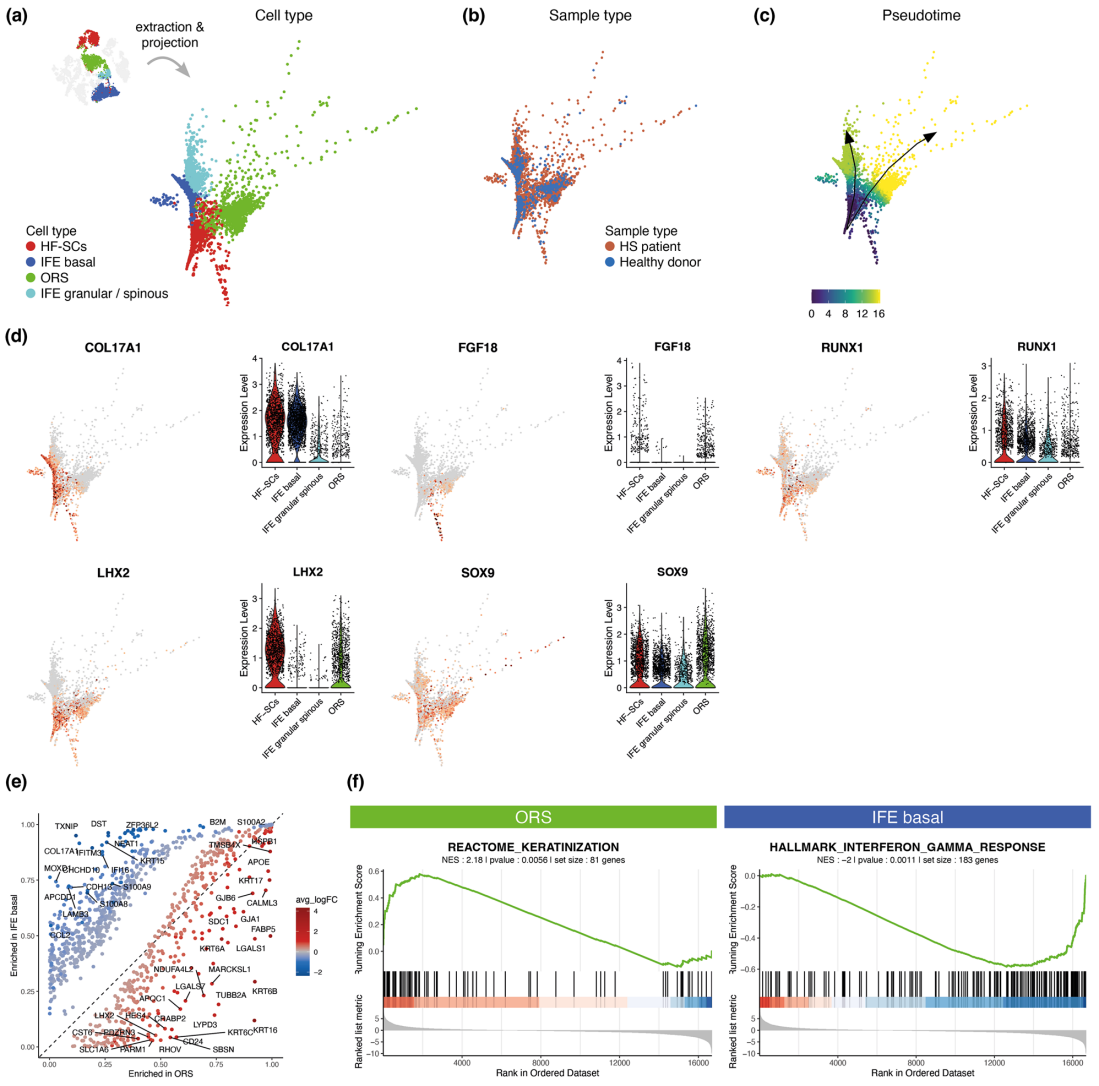
Non‐matrix cells from HF‐SCs follow two transcriptionally distinct lineages. (a–c) Diffusion map of merged HF‐SCs, IFE basal, IFE granular/spinous and ORS from our dataset (Figure 1), illustrated in the top left corner. Cells are coloured based on cell type annotation (a), sample type (b) or pseudotime (c). (d) Feature plots and violin plots showing expression levels of selected genes. Violin plots are split by cell types. (e) Representation of differentially expression genes between ORS and IFE basal from all samples. *X*‐ and *Y*‐axis respectively correspond to the proportion of cells within ORS and IFE basal cells with positive expression of the gene. Dashed line corresponds to genes expressed in same proportion of cells in ORS and IFE basal cells. One dot, corresponding to one gene, is coloured according to average log fold change (avg_logFC) between ORS and IFE basal cells. (f) GSEA plots made from average fold change of all genes, between ORS and IFE basal cells.

To investigate the differentiation dynamics of HF stem cells (HF‐SCs), we constructed a diffusion map based on integrated scRNA‐Seq data from both healthy donors and HS patients. This analysis revealed two conserved differentiation trajectories: one toward IFE basal and the other toward ORS (Figure 2a). These two distinct branches were consistently observed in both HS and healthy skin samples, indicating that this bifurcation represents a physiological feature of human HF‐SC differentiation (Figure 2b). Trajectory inference using the Slingshot package confirmed the existence of these two lineages, which was further validated using the TInGa algorithm (Figure 2c). Along the IFE branch, cells continued to differentiate into granular and spinous layers of the epidermis.

HF‐SCs expressed a core set of stemness‐associated genes, including key transcription factors such as SOX9, LHX2 and RUNX1, which help maintain stem cell quiescence and an undifferentiated state. Interestingly, their differentiated progeny retained subsets of these markers: IFE basal cells maintained SOX9, RUNX1 and COL17A1, while ORS cells retained SOX9, LHX2 and FGF18 (Figure 2d).

Transcriptomic profiling revealed striking molecular differences between these two epithelial lineages (Figure 2e). Gene Set Enrichment Analysis (GSEA) showed enrichment of keratinization‐related genes in the ORS branch, while IFE basal cells upregulated interferon response genes (Figure 2f). These lineage‐specific gene expression patterns were reproducible across the two additional datasets (Figure S2).

Altogether, our results suggest that HF‐SCs differentiate into two functionally distinct lineages: ORS cells contribute to structural processes like keratinization, while IFE basal cells may play a more prominent role in mediating inflammatory responses.

### Inflammatory shift in hair follicle epithelial lineages in HS

**FIGURE 3:**
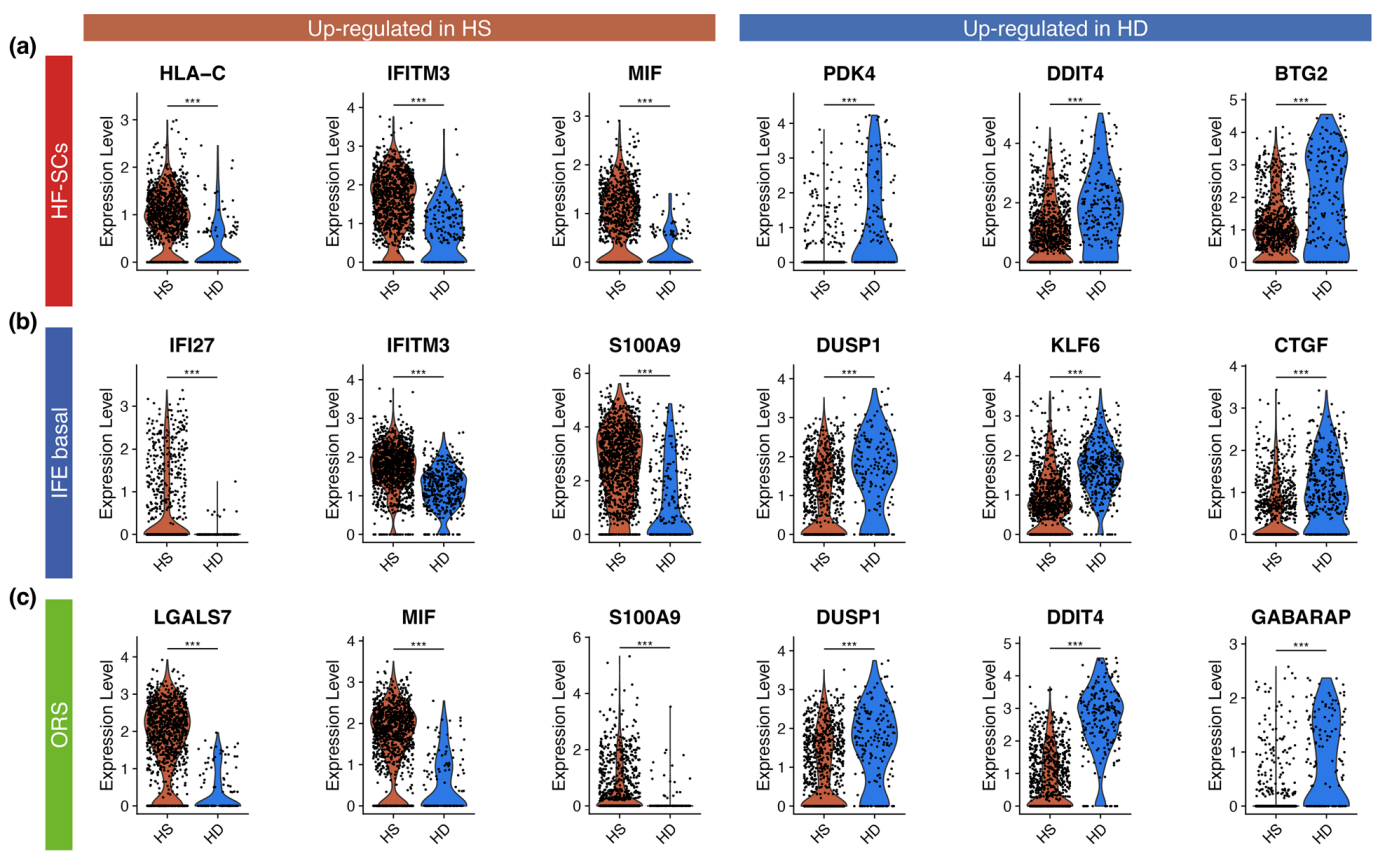
Non matrix cells show enrichment in inflammation markers in HS patients. Violin plots showing the expression level of differentially expressed genes between HS and HD, split by sample type, only within HF‐SCs (a), IFE basal (b) or ORS (c). Statistical *t*‐test *p*‐value, ^ns^>0.05, *<0.05 and >0.01, **<0.01 and >0.001 and ***< 0.001.

Differential expression analysis of HF‐SCs revealed fundamental transcriptomic changes between healthy donors and HS patients (Figure 3a). In HS patients, HF‐SCs displayed an inflammatory phenotype, as evidenced by an enrichment in HLA class I molecule, *MIF* and *IFITM3* (Figure 3a). Furthermore, HF‐SCs from HS patients were enriched for ribosomal genes suggesting a primed or activated transcriptional state. In contrast, HF‐SCs from healthy donors showed higher expression of genes involved in DNA repair, such as *DDIT4*, and the apoptosis pathway suggesting a more quiescent, homeostatic state (Figure 3a).

A similar shift toward inflammation was observed in IFE basal cells from HS patients, which exhibited increased expression of inflammatory mediators such as *CCL2* and *S100A9*, along with a Type I interferon signature (*IFI27, IFITM3*) (Figure 3b). Conversely, genes involved in cell cycle regulation (*DUSP1, KLF6*) and epithelial barrier integrity (*CTGF, CLDN1*) were downregulated in HS patients, potentially contributing to impaired epithelial homeostasis (Figure 3b).

In ORS cells, we likewise detected a shift toward inflammation and activation in HS patients. ORS cells overexpressed *MIF* and *S100A9*, consistent with immune activation (Figure 3c). Notably, we observed upregulation of *LGALS7* (galectin‐7), a lectin predominantly expressed in stratified epithelia,^10^ and *ARF5*, a small GTPase involved in splicing, ribosome synthesis and intracellular trafficking^11^ (Figure 3c). These changes suggest an altered differentiation state of ORS cells in HS. By contrast, ORS cells from healthy donors expressed higher levels of *DUSP1* and *DDIT4*, genes crucial for cell cycle control and cellular stress response.

Together, these results indicate that HF‐SCs, IFE basal and ORS cells in HS patients adopt a transcriptional programme characterized by inflammation, cellular activation and impaired cell cycle and adhesion processes—highlighting broad epithelial dysregulation in HS skin.

### Cytotoxic and inflammatory immune cell signatures emerge around hair follicles in HS

**FIGURE 4:**
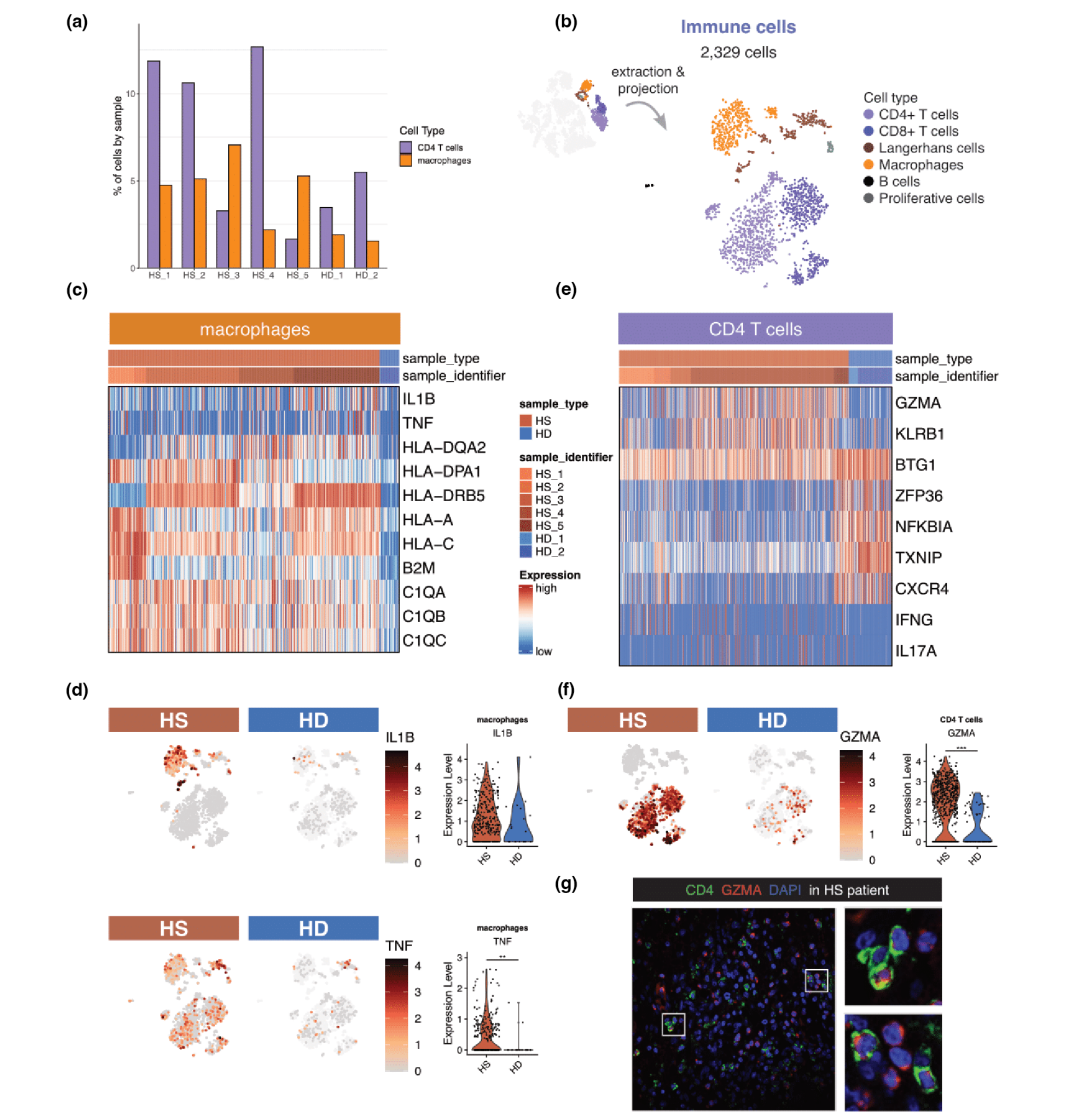
Immune cells in HS patients are activated compared to healthy donors. (a) Barplot showing the percentage of CD4^+^ T cells and macrophages in our dataset (Figure 1a), across sample identifiers. (b) tSNE plot showing 2329 immune cells extracted from our dataset. Cells are coloured based on cell type annotation. (c) Heatmap showing differentially expressed genes in macrophages, between HS and HD samples. (d) Feature plots and violin plots showing IL1B and TNF expression levels, split by sample type. On the tSNE, cells from complete immune cells dataset are shown as light background. The violin plots consider only macrophages. (e) Heatmap showing differentially expressed genes in CD4^+^ T cells, between HS and HD samples. Legend is shared with (c). (f) Feature plot and violin plot showing GZMA expression level, split by sample type. On the tSNE, cells from complete immune cells dataset are shown as light background. The violin plots consider only CD4^+^ T cells. (g) Section through hair follicles from a HS patient, immunolabelled from CD4, GZMA and DAPI. High magnification areas are denoted by square and represented on the right. Statistical *t*‐test *p*‐value, *<0.05, **<0.01 and ***< 0.001. For additional features in immune cells, refer to Figure S3.

We next investigated the immune cell populations present surrounding hair follicles. While healthy individuals exhibited minimal immune cell presence, HS patients displayed a marked infiltration of myeloid cells and/or T cells, accumulating around the hair follicles in the perilesional area (Figure 4a). To further investigate gene expression levels among immune cells, we generated a tSNE containing only immune cells from our dataset (Figure 4b).

Differential gene expression analysis revealed upregulation of MHC class I and II genes, as well as components of the complement pathway, in macrophages from HS patients compared to healthy donors (Figure 4c). Notably, the expression of *IL1B* and *TNF* levels was significantly elevated in HS macrophages (*p* = 0.058; *p* < 0.001, respectively) (Figure 4d), while *IL6* expression was undetectable (Figure S3A). These findings suggest the presence of a pro‐inflammatory macrophage phenotype in close proximity to hair follicles within perilesional regions.

CD4^+^ T cells exhibited a cytotoxic phenotype, characterized by elevated expression of *GZMA* and *KLRB1*, which was confirmed by immunofluorescence (Figure 4e–g). While nearly all CD4^+^ T cells expressed *GZMA*, *IFNG* and *IL17* expression varied markedly between individuals (Figure 4e). TCRα analysis showed no evidence for invariant TCR usage, indicating that CD4^+^ T cell infiltration is polyclonal (Figure S3E).

Furthermore, CD8^+^ T cells in HS patients exhibited significantly higher expression of *IFNG*, *GZMB* and *PRF1* compared to healthy donors, consistent with cytotoxic activity (Figure S3B–D). The generation of such cytotoxic T cells requires Type I interferon and IL12 signalling.^12^ Therefore, the presence of these cells further reinforces the pivotal role of interferon in HS pathogenesis, as we have previously demonstrated.^5^

### Self‐organizing map analysis of 49 HS patients reveals distinct clinical patterns linked to HF cell composition and pathophysiological mechanisms

Given the potential contribution of HF‐SC lineage divergence to HS heterogeneity, we sought to determine whether the IFE basal and ORS compartments are differentially enriched across patients. We hypothesized that this imbalance could reflect two distinct pathological mechanisms: one dominated by keratinization, linked to ORS enrichment and the other driven by inflammation, associated with IFE basal expansion. To investigate this, we analysed samples from 49 HS patients enrolled in the Fol‐Hydra cohort (Table 1). RNA was extracted from five freshly isolated hair follicles per patient and subjected to qPCR analysis to quantify markers of ORS, IFE basal and T cells, enabling us to infer the HF cellular composition in lesional skin. In parallel, and considering the central role of Nicastrin (NCSTN) in maintaining skin homeostasis—as well as the fact that approximately 37% of known HS‐associated variants map to this gene—we performed single nucleotide variant analysis of NCSTN in the same cohort. Notably, we identified two previously unreported variants, p.Leu30 and p.Gln83, both predicted to generate truncated proteins with potentially deleterious effects on function.

**TABLE 1:** Clinical characteristic of the 49 HS patients from the Fol‐HYDRA cohort.

Characteristic	*N*	Cluster A *N* = 8^a^	Cluster B *N* = 6^a^	Cluster C *N* = 9^a^	Cluster D *N* = 10^a^	Cluster E *N* = 16^a^	*p*‐value^b^
Clustering features							
Age	49	23.5 [20.5;25.3]	24.0 [20.8;25.8]	28.0 [26.0;35.0]	44.0 [39.0;51.0]	31.0 [23.0;40.8]	**<0.001**
Gender, females	49	5 (62.5%)	4 (66.7%)	2 (22.2%)	6 (60.0%)	12 (75.0%)	0.15
Age at HS diagnosis	48	18.0 [14.0;22.8]	20.0 [19.0;21.0]	20.0 [18.0;26.0]	34.0 [28.0;41.0]	27.5 [22.0;36.0]	**<0.001**
Age at symptoms onset	49	14.0 [14.0;15.5]	14.5 [14.0;15.8]	17.0 [16.0;18.0]	26.5 [25.0;30.0]	21.0 [17.0;23.8]	**<0.001**
HS familial history	38	3 (42.9%)	4 (66.7%)	0 (0.0%)	1 (12.5%)	5 (55.6%)	**0.021**
Smoking	35	1 (25.0%)	2 (50.0%)	5 (71.4%)	7 (77.8%)	9 (81.8%)	0.28
Body mass index (BMI)	49	25.0 [21.0;29.8]	27.5 [25.3;32.0]	25.0 [25.0;27.0]	30.5 [26.8;33.0]	23.5 [21.0;30.5]	0.27
LC1 Phenotype	49	7 (87.5%)	4 (66.7%)	3 (33.3%)	10 (100.0%)	11 (68.8%)	**0.015**
LC2 Phenotype	49	1 (12.5%)	2 (33.3%)	4 (44.4%)	0 (0.0%)	3 (18.8%)	0.13
LC3 Phenotype	49	0 (0.0%)	2 (33.3%)	4 (44.4%)	2 (20.0%)	2 (12.5%)	0.15
Pilonidal sinus	49	0 (0.0%)	2 (33.3%)	6 (66.7%)	1 (10.0%)	3 (18.8%)	**0.012**
Inflammatory nodule	49	6 (75.0%)	6 (100.0%)	6 (66.7%)	9 (90.0%)	1 (6.3%)	**<0.001**
Non‐inflammatory nodule	49	5 (62.5%)	3 (50.0%)	6 (66.7%)	6 (60.0%)	8 (50.0%)	0.93
Abscess	49	0 (0.0%)	1 (16.7%)	4 (44.4%)	1 (10.0%)	1 (6.3%)	0.076
Open comedone	49	2 (25.0%)	6 (100.0%)	5 (55.6%)	8 (80.0%)	4 (25.0%)	**0.002**
Bridge scar	49	2 (25.0%)	1 (16.7%)	6 (66.7%)	4 (40.0%)	0 (0.0%)	**0.002**
Cookie cutter scar	49	3 (37.5%)	4 (66.7%)	3 (33.3%)	6 (60.0%)	5 (31.3%)	0.44
Ice‐pick scar	49	2 (25.0%)	4 (66.7%)	6 (66.7%)	4 (40.0%)	3 (18.8%)	0.084
Draining fistula	49	0 (0.0%)	0 (0.0%)	5 (55.6%)	6 (60.0%)	1 (6.3%)	**<0.001**
Non‐draining fistula	49	2 (25.0%)	0 (0.0%)	5 (55.6%)	7 (70.0%)	1 (6.3%)	**0.001**
Papule	49	1 (12.5%)	1 (16.7%)	4 (44.4%)	2 (20.0%)	5 (31.3%)	0.63
Folliculitis	49	0 (0.0%)	1 (16.7%)	7 (77.8%)	0 (0.0%)	5 (31.3%)	**<0.001**
Continuous damage	49	3 (37.5%)	5 (83.3%)	9 (100.0%)	6 (60.0%)	4 (25.0%)	**0.001**
HS‐PGA score	49	2.00 [1.75;2.25]	3.00 [2.25;3.00]	3.00 [3.00;3.00]	2.00 [2.00;3.00]	1.00 [0.00;1.00]	**<0.001**
Dissecting cellulitis	49	0 (0.0%)	0 (0.0%)	1 (11.1%)	0 (0.0%)	0 (0.0%)	0.47
Folliculitis decalvans	49	0 (0.0%)	0 (0.0%)	2 (22.2%)	0 (0.0%)	0 (0.0%)	0.067
Keloidal acne	49	1 (12.5%)	0 (0.0%)	0 (0.0%)	0 (0.0%)	0 (0.0%)	0.29
Illustrative features							
NCSTN variants	49	0 (0.0%)	2 (33.3%)	0 (0.0%)	0 (0.0%)	0 (0.0%)	**0.033**
ORS specific gene	49	164 [69.0;260]	387 [294;584]	364 [331;667]	407 [315;535]	416 [317;464]	**0.023**
IFE basal ‐specific gene	49	61.1 [44.8;64.5]	57.8 [47.5;136]	65.2 [36.5;74.2]	67.5 [57.9;89.4]	30.5 [23.8;43.8]	**0.033**
T cells‐specific gene	49	4.18 [2.24;6.5]	7.5 [2.80;12.5]	2.29 [1.56;5.1]	2.57 [1.86;4.63]	2.06 [1.04;2.50]	0.068

*Note*: Bolded *p*‐values indicate statistical significance at the *p*<0.05 level.

^a^ Median [25%;75%]; *n* (%).

^b^ Kruskal–Wallis rank sum test; Fisher's exact test.

**FIGURE 5:**
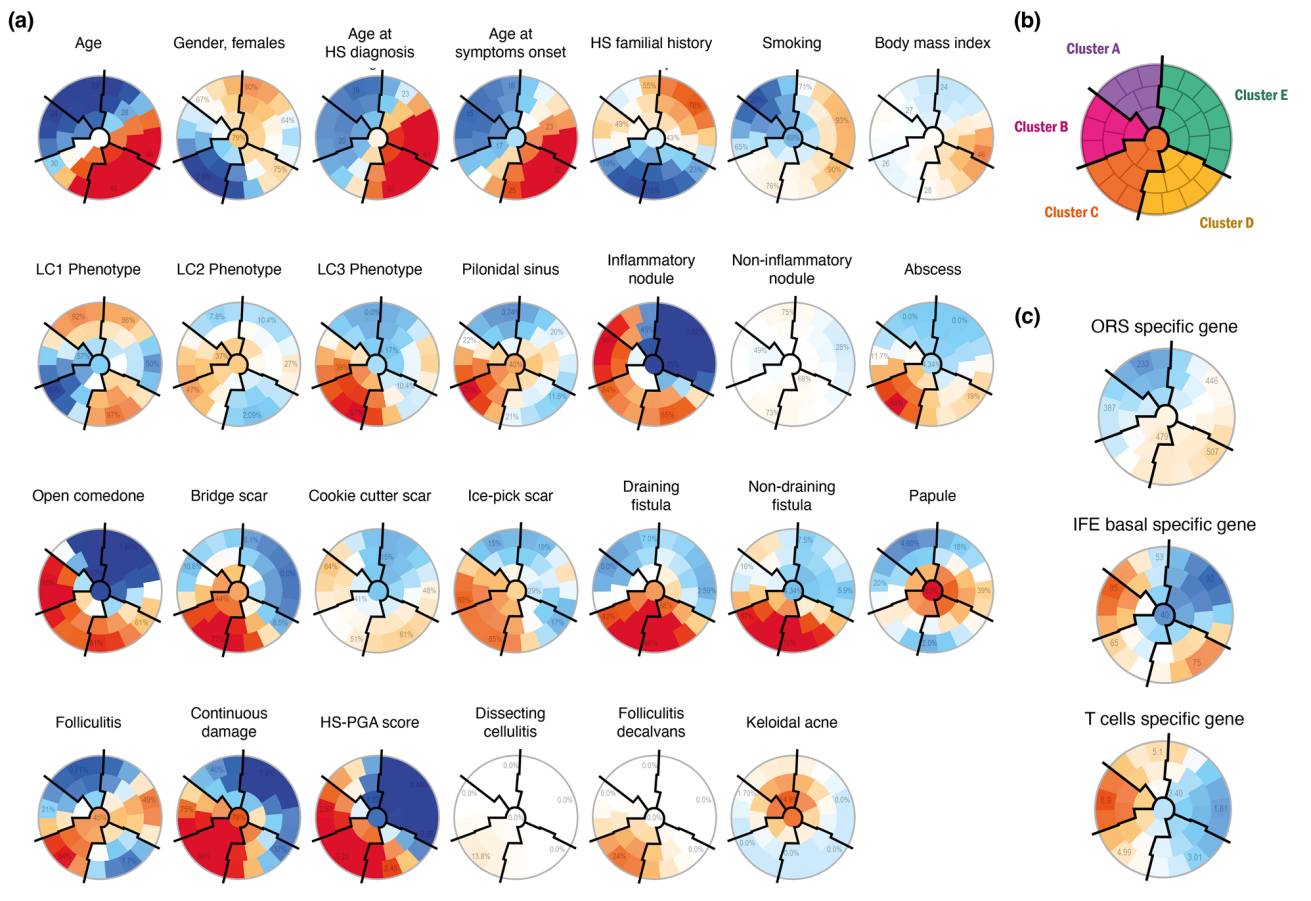
Clustering analysis of HS patients based on the self‐organizing maps (SOMs) methodology. (a) Unsupervised analysis by SOM placed all HS patients identified as globally similar for age, gender age at HS diagnosis, HS familial history and cigarette smoking within 1 of 40 small groupings (districts) throughout the maps. The more individuals resemble in terms of phenotyping, the closer they are placed on the map. Each individual map shows the mean values per district for each characteristic, blue colours indicating the lowest average values and red colours the highest, with detailed numbers written for a selection of representative districts in each SOM. (b) Close districts were combined to provide five suitable clusters of HS patients, labelled A to E. The clusters boundaries are delimited by solid black lines. (c) Representation of gene expression levels on the SOMs. These values were not taken into account while running the algorithm.

To address clinical heterogeneity, we first performed an unsupervised classification of patients based solely on clinical parameters, independently of qPCR results or NCSTN genotyping. Using self‐organizing maps (SOMs), we clustered patients according to their clinical features, revealing polarized distributions and five distinct clinical clusters (Figure 5a,b). Cluster boundaries are indicated by solid black lines, and a summary of clinical characteristics is provided in Table 1.

Patients in Cluster A (*n* = 8) and Cluster B (*n* = 6) comprised predominantly young, non‐smoking women with early disease onset. The key difference between these clusters lies in their clinical presentation: in Cluster A, 7 out of 8 patients exhibited an LC1 phenotype, whereas all patients in Cluster B presented with open comedones (100%), ice‐pick scars (66.6%) and continuous damage (83.3%). Additionally, 2 out of 6 patients in Cluster B had a pilonidal sinus, and notably, both patients with NCSTN variants belonged to this cluster. Strikingly, we observed a difference in HF composition, with an enrichment of IFE‐associated gene expression in Cluster B. Both clusters A and B showed an infiltration of T cells (Figure 5a,b).

Cluster C (*n* = 9) included mostly male smokers, with early disease onset and no family history of HS. Only one patient exhibited an LC1 phenotype. Half of the patients presented with fistulas and continuous tissue damage, alongside the highest Hidradenitis Suppurativa Physician Global Assessment (HS‐PGA) scores. This cluster showed co‐enrichment of IFE basal and ORS gene expression but lacked T‐cell infiltration.

Cluster D (*n* = 10) consisted of the oldest patients, with the highest BMI and chronic disease features, including varied scar types and fistulas. Similar to Cluster C, they showed dual enrichment in IFE basal and ORS markers without T‐cell infiltration.

Cluster E (*n* = 16) comprised mostly women with an LC1 phenotype, but with distinct demographics: older age, history of smoking, normal BMI and later disease onset. A significant proportion of patients had a family history of HS, and their nodules lacked inflammatory characteristics (Figure 5a,b). The HF cells in this cluster were enriched for ORS‐associated gene expression, again without T‐cell infiltration (Table 1).

These results demonstrated a correlation between the subtypes identified by distinct clinical features and HF cell composition. In conclusion, these data suggest that the clinical features arise from three distinct pathophysiological processes: (i) a process in which inflammation is clearly predominant (Cluster A), (ii) a process in which keratinization occurs without inflammation (Cluster E) and (iii) a process in which the two mechanisms are intertwined (Cluster B‐C‐D).

## DISCUSSION

Our work establishes a comprehensive framework for understanding how HF‐SCs contribute to HS pathogenesis. In healthy skin, HF‐SCs follow two conserved trajectories toward basal IFE or ORS, both essential for follicular homeostasis. IFE basal cells maintain epidermal renewal and barrier function, whereas ORS cells provide structural keratinization. In this setting, HF‐SCs remain largely quiescent and non‐inflammatory. In HS, this balance is disrupted: HF‐SCs, IFE and ORS compartments acquire a pro‐inflammatory profile, characterized by interferon response genes, S100 proteins and HLA expression, accompanied by macrophage infiltration producing IL‐1β and TNF and the accumulation of cytotoxic CD4^+^ and CD8^+^ T cells. These findings highlight the follicular epithelium not only as a structural target but also as an initiator of innate immune activation.

By integrating molecular and clinical data, we further demonstrate that the relative enrichment of IFE versus ORS lineages defines three endotypes—inflammatory, keratinizing and mixed. This framework refines the current concept of HS as an autoinflammatory keratinization disorder.^4^, ^6^ Zouboulis et al.^13^ emphasized epithelial dysfunction and innate immunity as the two molecular pillars of the disease and Dajnoki et al^14^ have provided evidence that the trigger of inflammatory signalling is localized in the epidermis. Our study pinpoints HF‐SC fate imbalance as a mechanistic substrate that underlies both processes. This explains why some patients predominantly present with inflammatory nodules, whereas others develop keratinizing and fistulizing disease. Complementing this model, Li et al.^15^ identified apocrine gland damage and keratin release as early epithelial events priming innate immune activation. Together, these findings converge on the concept that abnormal epithelial compartments—HF‐SCs, ORS and apocrine glands—are active drivers of innate immunity and chronic inflammation.

From a therapeutic perspective, our data suggest that treatment strategies in HS could be refined by aligning interventions with epithelial endotypes, complementing the approach of the recently published European S2k guidelines.^16^ These guidelines distinguish inflammatory forms, graded by IHS4, from non‐inflammatory forms, classified by Hurley staging for surgery, and emphasize combined medical and surgical management. Building on this, we propose a practical stratification: inflammatory endotypes would benefit from anti‐inflammatory strategies such as oral tetracyclines or clindamycin–rifampicin, with escalation to biologics (adalimumab, secukinumab, bimekizumab) in moderate‐to‐severe disease; keratinizing endotypes would be primarily managed by surgery, complemented by medical care to reduce flares; mixed endotypes, which are often refractory, may require novel therapies targeting both epithelial hyperproliferation and innate immune activation. While conceptual, this scheme illustrates how epithelial biology could inform precision medicine in HS.

Our study has several limitations. Although we validated endotype signatures in a sizeable clinical cohort (Fol‐Hydra, *n* = 49), treatment responses were not longitudinally assessed, which prevents direct correlation between epithelial composition and therapeutic outcomes. Moreover, our analysis was cross‐sectional, and it remains unclear whether patients transition between endotypes during disease evolution. Finally, functional studies are still required to experimentally demonstrate that HF‐SC fate imbalance can initiate innate immune activation and drive the observed clinical phenotypes.

Future work addressing these limitations will be crucial to validate our model. Prospective, longitudinal studies combining molecular profiling with therapeutic outcomes will be needed to determine whether epithelial‐based stratification can be translated into clinical practice. If confirmed, such an approach would provide the basis for a true precision medicine framework in HS, where treatment choices are informed by epithelial endotype in addition to clinical severity.

## AUTHOR CONTRIBUTIONS

Conceptualization (AO, YL, PT, EA, SH); data acquisition (FJL, PLC, FC, RA, CBo, SA, ES, Cbe, JFD); analysis (AO, FJL, FC, EA, KM, EB); interpretation (AO, FJL, EA, SH); visualization (AO, EA, SH); writing original draft (AO, SH); writing and review (AO, FJL, PLC, ES, PW, VG, PT, EA, KM, JFD, EB, SH); project administration (PW, VG, YL, PT, EA, SH); funding acquisition (SH).

## FUNDING INFORMATION

This work was supported by the Institut National de la Santé et de la Recherche Médicale (INSERM), the University Paris Est Créteil (UPEC). It has received financial support from the French ‘Agence Nationale de la Recherche’ (ANR) under project ANR‐20‐CE17‐0019 and ‘Société Française de Dermatologie et de Pathologie Sexuellement Transmissible’.

## CONFLICT OF INTEREST STATEMENT

All authors have declared that no conflict of interest exists.

## ETHICAL APPROVAL

This monocentric, prospective study was conducted in accordance with the Declaration of Helsinki and approved by the appropriate ethics committee (CPP North‐West IV: 2021‐A02352‐39, 13/01/2022 for the clinical study and 20.12.11.69413 for the scRNA‐Seq analysis).

## ETHICS STATEMENT

All patients gave written informed consent before study enrolment.

## Supporting information


Table S1.

Table S2.

Figure S1.

Figure S2.

Figure S3.

Appendix S1.

## Data Availability

The single‐cell RNA Sequencing data underlying this article are available in Gene Expression Omnibus and can be accessed with the accession number GSE273109. Their computational processing is accessible at https://github.com/audrey‐onfroy/Onfroy_et_al_scRNASeq_HS_2025. The specific version of the analysis presented here is stored on Zenodo under record ID 15586365. The Singularity container to render the notebooks is accessible on Zenodo, with record ID 15512051 (https://zenodo.org/records/15512051). The record also contains a table summarizing the version of all the R packages involved in the study.

## References

[1] Matusiak Ł . Profound consequences of hidradenitis suppurativa: a review. Br J Dermatol. 2020;183:e171–e177. 29744872 10.1111/bjd.16603

[2] Zouboulis CC , Benhadou F , Byrd AS , Chandran NS , Giamarellos‐Bourboulis EJ , Fabbrocini G , et al. What causes hidradenitis suppurativa? 15 years after. Exp Dermatol. 2020;29:1154–1170. 33058306 10.1111/exd.14214

[3] Canoui‐Poitrine F , Le Thuaut A , Revuz JE , Viallette C , Gabison G , Poli F , et al. Identification of three hidradenitis Suppurativa phenotypes: latent class analysis of a cross‐sectional study. J Invest Dermatol. 2013;133:1506–1511. 23235532 10.1038/jid.2012.472

[4] Nomura T . Hidradenitis suppurativa as a potential subtype of autoinflammatory keratinization disease. Front Immunol. 2020;11:847. 32508815 10.3389/fimmu.2020.00847 PMC7251184

[5] Orvain C , Lin Y‐L , Jean‐Louis F , Hocini H , Hersant B , Bennasser Y , et al. Hair follicle stem cell replication stress drives IFI16/STING‐dependent inflammation in hidradenitis suppurativa. J Clin Invest. 2020;130:3777–3790. 32240121 10.1172/JCI131180 PMC7324185

[6] Frew JW . Hidradenitis suppurativa is an autoinflammatory keratinization disease: a review of the clinical, histologic, and molecular evidence. JAAD Int. 2020;1:62–72. 34409324 10.1016/j.jdin.2020.05.005 PMC8361883

[7] Lowe MM , Naik HB , Clancy S , Pauli M , Smith KM , Bi Y , et al. Immunopathogenesis of hidradenitis suppurativa and response to anti‐TNF‐α therapy. JCI Insight. 2020;5:e139932. 32841223 10.1172/jci.insight.139932 PMC7566733

[8] Wu S , Yu Y , Liu C , Zhang X , Zhu P , Peng Y , et al. Single‐cell transcriptomics reveals lineage trajectory of human scalp hair follicle and informs mechanisms of hair graying. Cell Discov. 2022;8:49. 35606346 10.1038/s41421-022-00394-2 PMC9126928

[9] Takahashi R , Grzenda A , Allison TF , Rawnsley J , Balin SJ , Sabri S , et al. Defining transcriptional signatures of human hair follicle cell states. J Invest Dermatol. 2020;140:764–773. 31676413 10.1016/j.jid.2019.07.726 PMC7093259

[10] Gendronneau G , Sanii S , Dang T , Deshayes F , Delacour D , Pichard E , et al. Overexpression of galectin‐7 in mouse epidermis leads to loss of cell junctions and defective skin repair. PLoS One. 2015;10:e0119031. 25741714 10.1371/journal.pone.0119031 PMC4351092

[11] Li Q , Li F , Song X , Lu N , Jing X , Wen H , et al. Pan‐cancer analysis of ARFs family and ARF5 promoted the progression of hepatocellular carcinoma. Heliyon. 2024;10:e29099. 38617932 10.1016/j.heliyon.2024.e29099 PMC11015141

[12] Hua L , Yao S , Pham D , Jiang L , Wright J , Sawant D , et al. Cytokine‐dependent induction of CD4+ T cells with cytotoxic potential during influenza virus infection. J Virol. 2013;87:11884–11893. 23986597 10.1128/JVI.01461-13 PMC3807312

[13] Zouboulis CC , Nogueira da Costa A , Makrantonaki E , Hou XX , Almansouri D , Dudley JT , et al. Alterations in innate immunity and epithelial cell differentiation are the molecular pillars of hidradenitis suppurativa. J Eur Acad Dermatol Venereol. 2020;34:846–861. 31838778 10.1111/jdv.16147

[14] Dajnoki Z , Somogyi O , Medgyesi B , Jenei A , Szabó L , Gáspár K , et al. Primary alterations during the development of hidradenitis suppurativa. J Eur Acad Dermatol Venereol. 2022;36:462–471. 34724272 10.1111/jdv.17779 PMC9298903

[15] Li J , Li S , Zhang Q , Liang M , Chen X , Feng Y , et al. Apocrine gland damage and the release of specific keratins in early stage indicate the crucial involvement of apocrine glands in hidradenitis Suppurativa. J Invest Dermatol. 2025;145:1371–1384. 39547394 10.1016/j.jid.2024.09.021

[16] Zouboulis CC , Bechara FG , Benhadou F , Bettoli V , Bukvić Mokos Z , Del Marmol V , et al. European S2k guidelines for hidradenitis suppurativa/acne inversa part 2: treatment. J Eur Acad Dermatol Venereol. 2025;39:899–941. 39699926 10.1111/jdv.20472 PMC12023723

