# Supplementary material for: Hair follicle stem cell fate supports distinct clinical endotypes in hidradenitis suppurativa
Source: J Eur Acad Dermatol Venereol. 2025 Nov 6;40(3):473–83. doi: 10.1111/jdv.70152 (PMC12933698; doi:10.1111/jdv.70152)
Supplement: Supplementary file 1 — Table S1. [file JDV-40-473-s001.docx]

| Patient | Sex | Age (years) | Phenotype | Hurley Score | Sampling site |
| --- | --- | --- | --- | --- | --- |
| HS 1 | M | 44 | LC2/3 | III | axillary |
| HS 2 | M | 26 | LC1/3 | III | axillary |
| HS 3 | M | 28 | LC2 | II | pubis |
| HS 4 | F | 20 | LC1 | III | axillary |
| HS 5 | F | 21 | LC1 | III | axillary |
| HD 1 | M | 31 |  |  | pubis |
| HD 2 | F | ND |  |  | pubis |

Table S1: Clinical characteristics of patients (n=7) for scRNA Seq analysis

ND Not determined
