# Supplementary material for: Hair follicle stem cell fate supports distinct clinical endotypes in hidradenitis suppurativa
Source: J Eur Acad Dermatol Venereol. 2025 Nov 6;40(3):473–83. doi: 10.1111/jdv.70152 (PMC12933698; doi:10.1111/jdv.70152)
Supplement: Supplementary file 2 — Table S2. [file JDV-40-473-s005.docx]

| CD4 T cells | CD3D, CD3G, CD3E, CD52, IL32, TRBC1, CD4, COTL1, CD40LG, GPR183, TNFRSF1B, FYB1, LAT |
| --- | --- |
| CD8 T cells | CD3D, CD3G, CD3E, CD52, IL32, CTSW, NCR3, CD8A, NKG7, KLRC1, TRGC2, CCL5, ZNF683 |
| Langerhans cells | CD207, LST1, AIF1, CPVL, C15orf48, CD1A, CD52, CD1E, CD1C |
| macrophages | LST1, AIF1, C3, OLFML3, TREM2, A2M, MSR1, FCGR3A, VSIG4, AP003481.1 |
| B cells | CD79A, IGHG2, IGHG3, IGLC3, IGHG1, IGHM, MS4A1, IGLC2, IGHGP, BANK1, IGHG4, IGKC, TNFRSF13C, CD79B, KLF2, GZMB |
| cuticle | KRT32, KIF21A, CCND2, VCAN, CYSTM1, TESC, MT4, KRT35, DIAPH3, VSNL1, NOTCH1, SNCAIP, CADM1, OCA2, LAMP5 |
| cortex | KRTAP11-1, PRR9, C10orf99, CRYAB, HEPHL1, ALOX15B, EFHD1, KRT35, CALHM4, SPECC1, DSG4, DNASE1L2, TGM3, KRT31, KRT36, KIAA1211L |
| medulla | BAMBI, SLC7A8, EDNRA, ALDH1A3, IGFBP2, KRT25, KITLG, MSX2, SLC6A15, PRDM1 |
| ORS | KRT71, KRT27, KRT28, KRT25, CTSC, GATA3, KRT72, KRT73, KRT74, SCEL, GFRA3, POF1B, RDH12, LMO7, TCHH, FABP9 |
| proliferative | NUSAP1, PCLAF, TOP2A, MKI67, BIRC5, CENPF, TK1, CENPU, TYMS, MCM5, GINS2, MCM5, PCLAF, CDCA7, MCM3, TYMS, CLSPN, FEN1, FAM111B, CDT1 |
| HF-SCs | KRT15, DST, DIO2, TCEAL2, SFRP1, TNC, WIF1, FRZB, LHX2, SPRY1, COL1A2, ITGA6, SOX9, S100A6, ANGPTL7, LGR5, COL17A1 |
| IFE basal | GPX2, IMPA2, KRT15, DST, COL17A1, MOXD1, C19orf48, CHD7, ALDH3A1, AHNAK2, CDH13, LAMB3, CAVIN1, EHF, CEBPD, TP53AIP1 |
| IFE granular spinous | GPX2, IMPA2, DEFB1, LY6D, EHF, PDZK1IP1, S100A7, FGFBP1, LYPD3, CRABP2, SPINK5, KRT1, KRTDAP, CIDEA, SUSD4, FAM83A, TMEM45A |
| ORS | KRT16, KRT6B, KRT6C, NDUFA4L2, TM4SF1, APOC1, SLC1A6, CLCA2, RHOV, CBLN2, HES4, SMCO4, MGST1, LYPD3, MGP |
| melanocytes | DCT, KIT, MLANA, GPM6B, EDNRB, PMEL, IGFBP7, MITF, ZEB2, APOD |
| sebocytes | CLMP, GLDC, PPARG, ACSBG1, MGST1, SAA1, AR, APMAP |

Table S2: Gene markers for single-cell level cell type annotation of scRNA-Seq data.
