# Supplementary figures and images for: Hair follicle stem cell fate supports distinct clinical endotypes in hidradenitis suppurativa
Source: J Eur Acad Dermatol Venereol. 2025 Nov 6;40(3):473–83. doi: 10.1111/jdv.70152 (PMC12933698; doi:10.1111/jdv.70152)

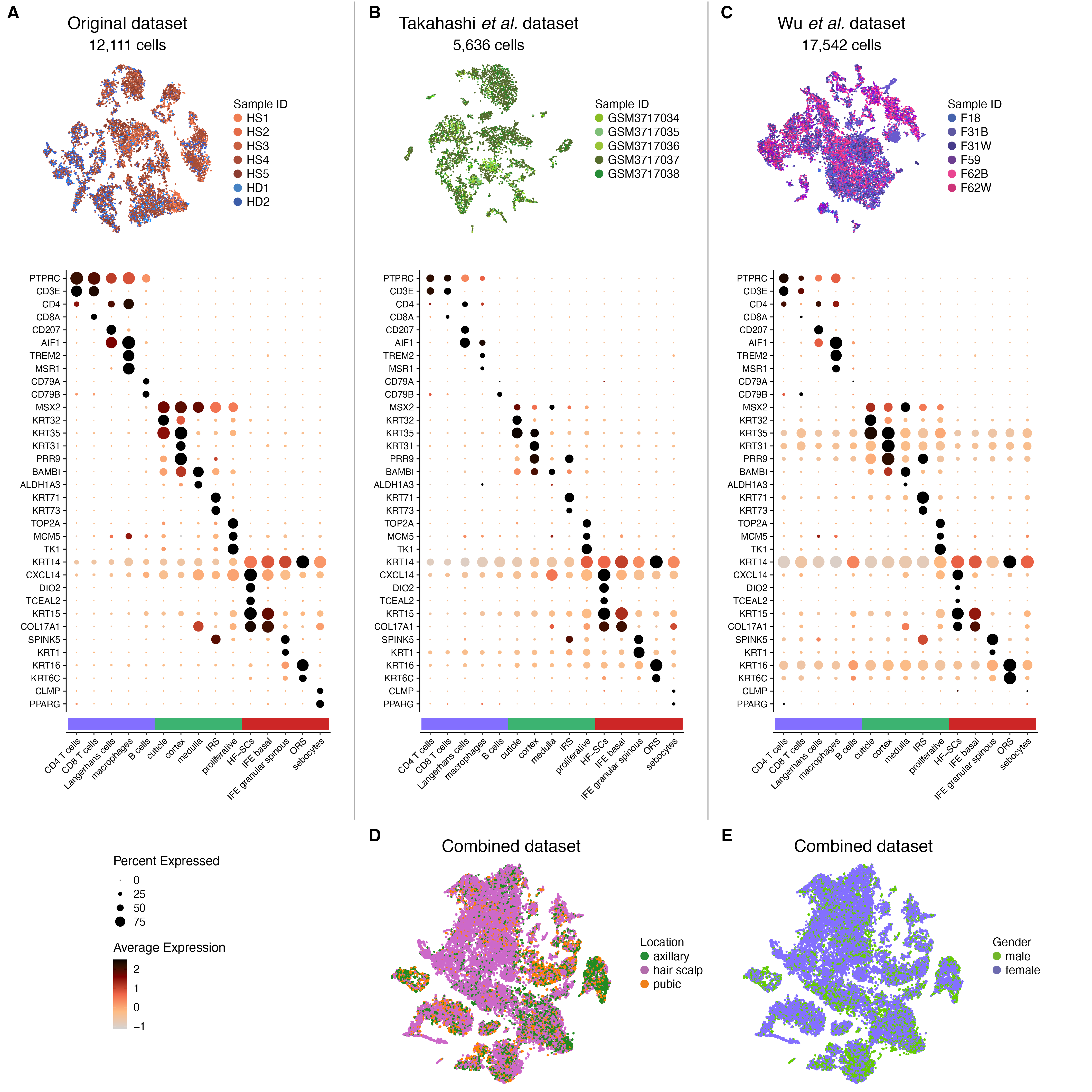

Supplement: Supplementary file 3 — Figure S1. [file JDV-40-473-s003.png]

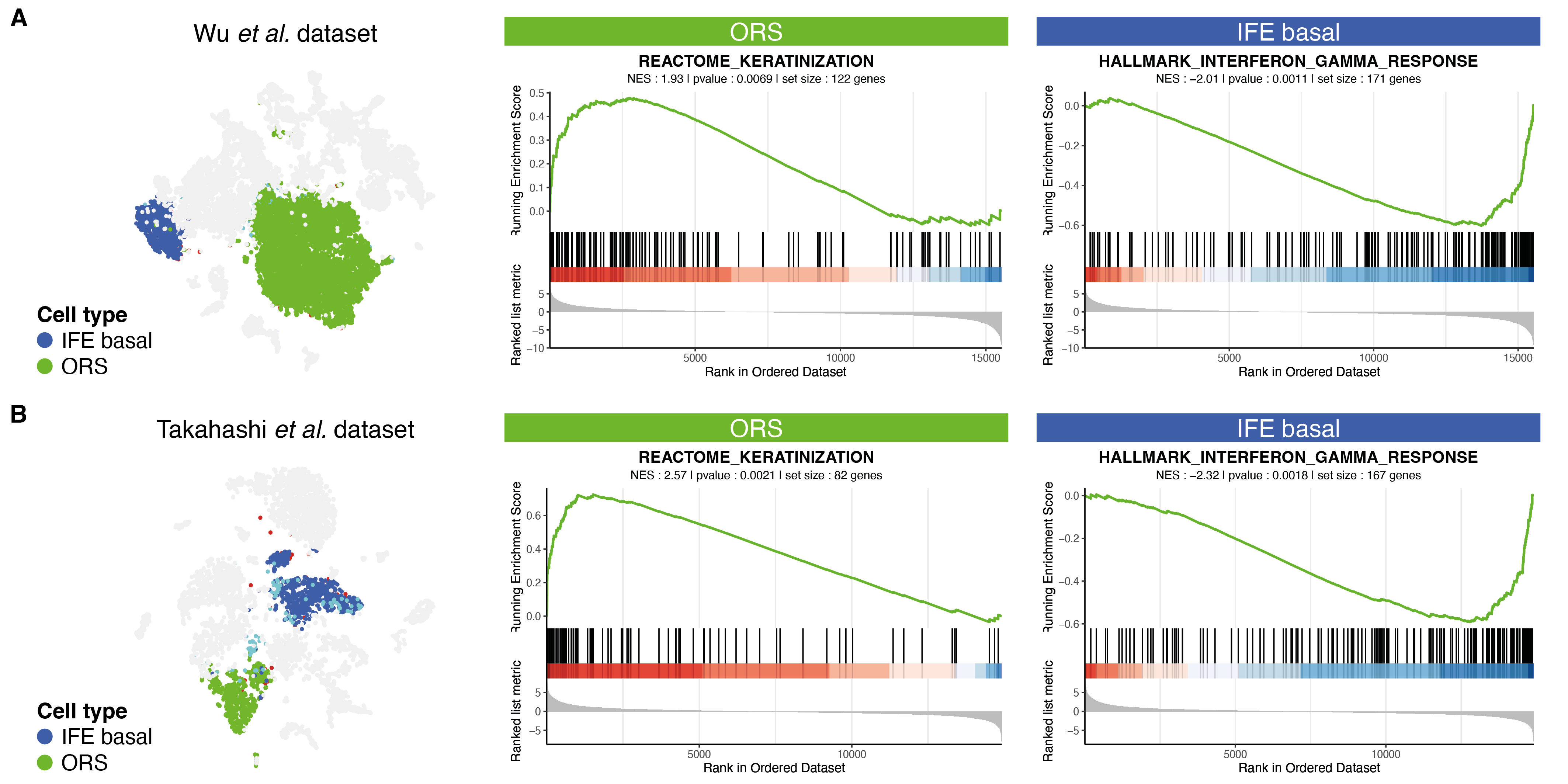

Supplement: Supplementary file 4 — Figure S2. [file JDV-40-473-s004.png]

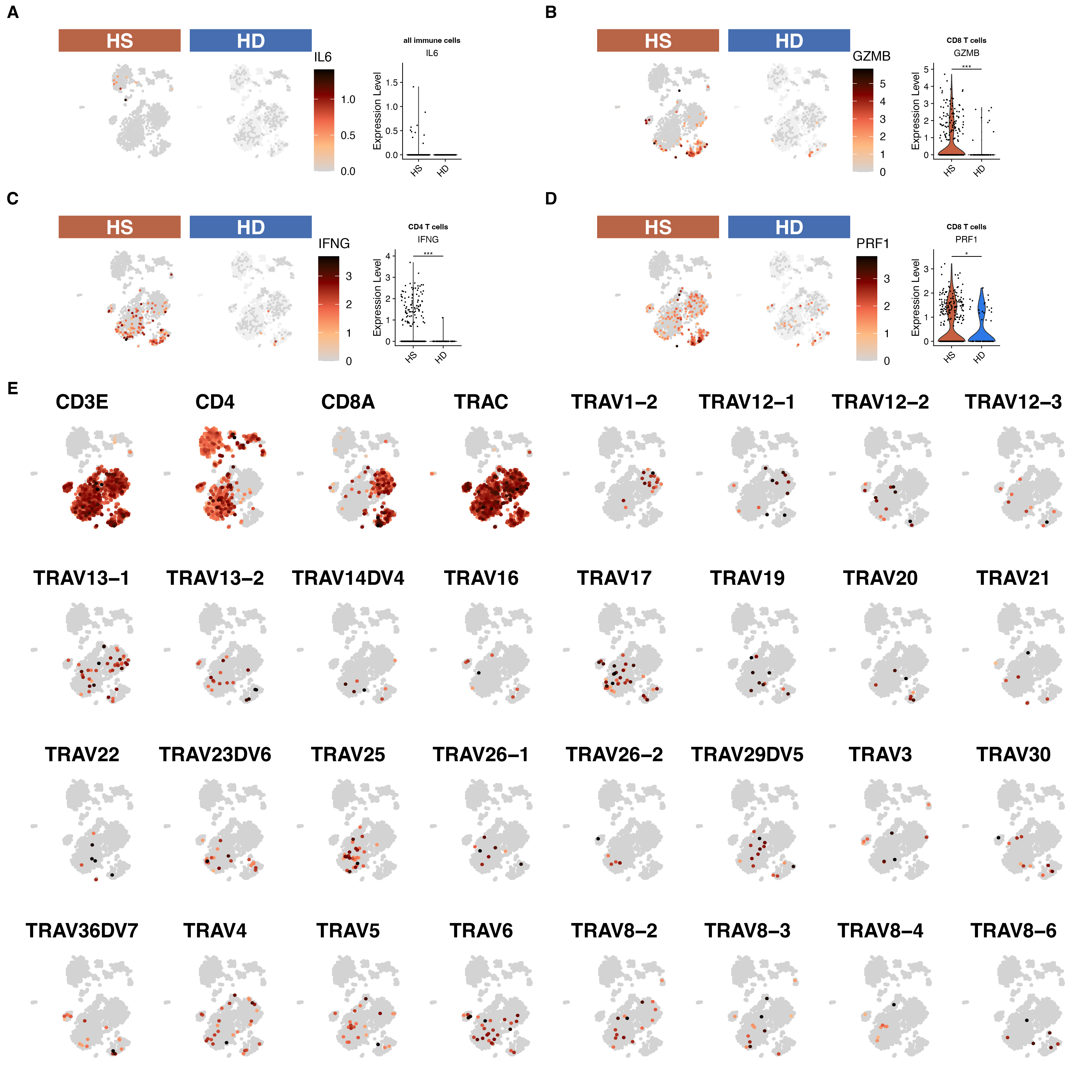

Supplement: Supplementary file 5 — Figure S3. [file JDV-40-473-s006.png]
