# Supplementary material for: Hair follicle stem cell fate supports distinct clinical endotypes in hidradenitis suppurativa
Source: J Eur Acad Dermatol Venereol. 2025 Nov 6;40(3):473–83. doi: 10.1111/jdv.70152 (PMC12933698; doi:10.1111/jdv.70152)
Supplement: Supplementary file 6 — Appendix S1. [file JDV-40-473-s002.docx]

**MATERIALS AND METHODS**

**Ethic of human samples**

This study was conducted on a cohort comprising men and women with HS. HS patients were recruited from the Fol-HYDRA study and enrolled from the dermatology department of the Henri Mondor hospital between June 2022 and December 2023. The Clinical Investigation Center performed skin biopsies. This monocentric, prospective study was conducted in accordance with the declaration of Helsinki and approved by the appropriate ethics committee (CPP North-West IV: 2021-A02352-39, 13/01/2022). All patients gave written informed consent before study enrolment.

For scRNA-Seq analysis, hair follicle samples were from discarded plastic surgery specimens from two healthy individuals and five HS patients (Table S1). This study was conducted in accordance with the declaration of Helsinki and was approved by the French institutional committee (reference N° 20.12.11.69413). The main inclusion criteria were 1) history of HS according to the diagnostic provided by the experimented investigator and 2) aged 18 years or older.

**Human HF separation**

Human samples were digested in 1 U/mL Dispase (Stemcell Technologies) overnight at +4°C. Individual hair follicles were pulled out using forceps. The isolated hair follicles were digested in TrypLE Express 1X (Gibco) for 30 minutes at room temperature, washed in DMEM + 10% FBS, and filtered through a 100μm cell strainer.

**Single-cell RNA sequencing**

HF cells were sorted (BD InfluxTM Cell Sorter) using a nuclear labelling DRAQ7TM Far-Red Fluorescent Live-Cell Impermeant DNA Dye (Abcam, ref: ab109202). Cells were collected in 0.04% BSA-RPMI solution.

For each individual, 25,000 cells were loaded into one channel of the Chromium system using the V3.1 single-cell reagent kit (10X Genomics). Following capture and lysis, cDNAs were synthesized, and then amplified by PCR for 12 cycles. The amplified cDNAs were used to generate Illumina sequencing libraries that were sequenced on one flow cell Nextseq500 Illumina.

**Analysis of scRNA-Seq data**

Sequencing files were processed using 10x Genomics Cell Ranger 3.1.0. Reads were mapped on the GRCh37 (hg19) transcriptome. Raw count matrices were processed up to the figures presented here using RStudio, with R Markdown to generate fully traceable notebooks. To ensure version stability, a Singularity container containing R version 3.6.3 and all packages of interest was developed and used to compile notebooks. HTML reports, including codes and figures, are available in a Github repository as an intuitive website (see the Code and Data Availability section).

Individual datasets, associated with a single patient sample, were analyzed individually from the raw count matrix using Seurat V3 package (1). Cells having less than 500 genes were filtered out. Doublet cells were removed using scDblFinder tool (2) and scds in hybrid mode (3). Then, cells having more than 20% of UMI related to mitochondrial genes or more than 50% related to ribosomal genes were filtered out. The UMI count matrix for remaining cells was normalized using LogNormalize method implemented in Seurat::NormalizeData function. Single cells were annotated for cell type using a modified version of Seurat::AddModuleScore and cell type-specific marker gene sets in Table S2. According to previous publications associated with scRNA-Seq from skin(4–6), they encompass CD4+ T cells, CD8+ T cells, Langerhans cells, macrophages, B cells, cuticle, cortex, medulla, IRS (inner root sheath), proliferative cells, ORS (outer root sheath), IFE (interfollicular epidermis), HF-SC (hair follicle stem cell), sebocytes and melanocytes. To smoothen eventual mis-annotation, single cell level annotation was grouped by cluster. Clustering was generated as follows. First, 3000 highly variable features (HVFs) were identified using Seurat::FindVariableFeatures. Then, we generated a principal component analysis (PCA) with 100 components, from those 3000 HVFs, using Seurat::RunPCA. Distances between cells were computed using top 20 components of the PCA, with Seurat::FindNeighbors. Finally, a clustering was generated by Seurat::FindClusters with resolution 2. The cluster annotation was defined as the most represented cell type by cluster.

To build the main “atlas” dataset (Figure 1A), melanocytes clusters were removed in individual datasets prior to combination. Dataset combination was made using the base::merge function. For the populations-specific datasets associated with non-matrix cells, IFE basal and ORS, and immune cells, clusters of interest were selected, in the atlas dataset, based on their annotation. Genes expressed in less than 5 cells were removed. Merged count matrices were normalized using LogNormalize method and a 100 components PCA was made using the scaled expression of 2000 HVFs. Sample-specific effect was removed using harmony::RunHarmony on the PCA. The tSNE was built using Seurat::RunTSNE function.

For the dataset containing non-matrix cells, a diffusion map was used to generate a projection representative of transcriptomic changes between cells, using the implementation from destiny package (7). Trajectory inference was made using two distinct methods: slingshot (8) and TInGa (9), using cells in HF-SCs population as trajectory root, and the harmonized PCA as input. Slingshot output 7 lineages, either with dead end within HF-SC population, or toward IFE basal cells and ORS cells. TInGa can infer branching trajectory, thus was used for visualization purpose. Default parameters, except max_nodes set to 10, were used. Differential expression was conducted using Seurat::FindMarkers. Heatmaps were made using ComplexHeatmap package (10). Functional enrichment analyses were conducted using msigdbr package for the gene sets database, and clusterProfiler package for the analyses (11). Gene sets score were computed using the Seurat::AddModuleScore function. When comparing the expression levels of scores between two populations, t-test implemented in the stats::t.test function was used.

To validate our findings, the same processing steps were applied to the dataset from Wu *et al.* and Takahashi *et al.*. For the former, input data were the FASTQ files, downloaded at project number OEP002321 on biosino.org portal. For the latter, the analysis was conducted from the count matrices downloaded at the accession number GSE129611 on Gene Set Omnibus portal. The detailed parameter setting are accessible in the compiled HTML files obtained from the R Markdown notebooks (see the Code and Data Availability section).

**RNA extraction and Real time Quantitative PCR**

RNA extraction was performed according to the manufacturer’s protocol (RNeasy Micro Kit, QIAGEN Inc.). RNA was converted to cDNA with QuantiTect reverse transcription Kit (QIAGEN). Quantitative PCRs were performed using the Brilliant II SYBR GREEN QPCR Master Mix kit (Agilent Technologies) for the expression of three genes specific for T cells, IFE basal cells and ORS cells. Expression of the gene OAZ1 was used as a reference and the relative levels of each gene were calculated using the 2ΔΔCT method.

**Whole Genome Sequencing**

Whole-genome sequencing was performed at the Centre National de Recherche en Génomique Humaine. After a complete quality control, genomic DNA (1µg) was used to prepare a library for whole genome sequencing, using the Illumina TruSeq DNA PCR-Free Library Preparation Kit (Illumina Inc., CA, USA), according to the manufacturer's instructions. After quality control and normalisation, qualified libraries have been sequenced on a NovaSeqX+ platform from Illumina (Illumina Inc., CA, USA), as paired-end 150 bp reads. Samples were pooled on a NovaSeqX+ 25B flowcell in order to reach an average sequencing depth of 30X. Sequence quality parameters were assessed throughout the sequencing run and standard bioinformatics analysis of sequencing data was based on the Illumina pipeline to generate FASTQ files for each sample.

**Variant calling, annotation and analysis**

The individual .g.vcf files were obtained through an in-house pipeline using the following tools: bwa-mem (v. 2.2.1), PicardTools (v. 2.26.9), Samtools (v. 1.16), Sambamba (v. 0.8.1), GATK (v. 4.2.3.0), and bedtools (v. 2.30.0). The alignment was performed on the reference genome, GRCh38. The .g.vcf files were aggregated and then transformed into .vcf files using the GATK CombineGVCFs and GenotypeGVCFs modules. The variants were filtered according to the commonly used criteria: (1) for SNVs: QD < 2, QUAL < 30, SOR > 3, FS > 60, MQ < 40, MQRankSum < −12.5, and ReadPosRankSum < −8; (2) for Indels: QD < 2, QUAL < 30, FS > 200, and ReadPosRankSum < −20. We annotated our variants with the SnpEff suite (v. 4.3.t) and the SnpEff variant database (v. 5.1). For this work, only the MANE NCSTN transcript (ENST00000294785) biallelic variants with a strong impact (the most deleterious) have been retained. Variant allele frequencies are provided by gnomAD (v. 4.1).

**Clustering analysis of patients**

Clustering analyses consisted of using the self-organizing maps (SOMs) algorithm developed by Kohonen. In a nutshell, the SOMs algorithm assigns each individual to a specific area on the map based on their characteristics, placing similar individuals in proximity and distinct ones in remote locations, thus allowing to draw visual comparisons of unique or overlapping patient characteristics and disease subtypes. The SOMs were constructed by applying the approach developed within the Numero package framework for the R statistical platform 1) building the SOMs with statistical verification of the robustness of the contrasts observed by permutation tests and 2) determining suitable groupings based on the direct visualization of data patterns and key characteristics of the dataset.

For illustrative purposes, Gabriel’s biplots were plotted to project the patients along the principal components axes from mixed principal component analysis according to their individual characteristics, colouring patients according to their final diagnosis or cluster and thus allowing for direct visual assessment of the discriminative ability of each subgrouping.

For descriptive statistics, categorical variables are expressed as proportions (%), quantitative variables as means (±standard deviation [SD]) or medians (interquartile range [IQR]), as appropriate. Biological parameters were log-transformed due to the non-normality of their distribution. We compared groups from clustering by means of one-way ANOVA or Kruskal-Wallis tests for continuous variables and chi-square tests or Fisher’s exact tests for categorical variables. A p-value < 0.05 was considered significant. Descriptive statistics and between-clusters comparisons were realized using Stata software 15.1 (StataCorp, Tx, USA), and R 3.4.3 (R Foundation, Vienna, Austria; pca2d, Numero packages) for clustering analyses and visualizations.

**REFERENCES**

1. Stuart T, Butler A, Hoffman P, Hafemeister C, Papalexi E, Mauck WM, et al. Comprehensive Integration of Single-Cell Data. Cell. 13 juin 2019;177(7):1888-1902.e21.

2. Germain PL, Lun A, Garcia Meixide C, Macnair W, Robinson MD. Doublet identification in single-cell sequencing data using scDblFinder. F1000Res. 2021;10:979.

3. Bais AS, Kostka D. scds: computational annotation of doublets in single-cell RNA sequencing data. Bioinformatics. 15 févr 2020;36(4):1150‑8.

4. Wu S, Yu Y, Liu C, Zhang X, Zhu P, Peng Y, et al. Single-cell transcriptomics reveals lineage trajectory of human scalp hair follicle and informs mechanisms of hair graying. Cell Discov. 24 mai 2022;8(1):49.

5. Takahashi R, Grzenda A, Allison TF, Rawnsley J, Balin SJ, Sabri S, et al. Defining Transcriptional Signatures of Human Hair Follicle Cell States. J Invest Dermatol. avr 2020;140(4):764-773.e4.

6. Joost S, Annusver K, Jacob T, Sun X, Dalessandri T, Sivan U, et al. The Molecular Anatomy of Mouse Skin during Hair Growth and Rest. Cell Stem Cell. 5 mars 2020;26(3):441-457.e7.

7. Angerer P, Haghverdi L, Büttner M, Theis FJ, Marr C, Buettner F. destiny: diffusion maps for large-scale single-cell data in R. Bioinformatics. 15 avr 2016;32(8):1241‑3.

8. Street K, Risso D, Fletcher RB, Das D, Ngai J, Yosef N, et al. Slingshot: cell lineage and pseudotime inference for single-cell transcriptomics. BMC Genomics. 19 juin 2018;19(1):477.

9. Todorov H, Cannoodt R, Saelens W, Saeys Y. TinGa: fast and flexible trajectory inference with Growing Neural Gas. Bioinformatics. 1 juill 2020;36(Suppl_1):i66‑74.

10. Gu Z, Eils R, Schlesner M. Complex heatmaps reveal patterns and correlations in multidimensional genomic data. Bioinformatics. 15 sept 2016;32(18):2847‑9.

11. Yu G, Wang LG, Han Y, He QY. clusterProfiler: an R package for comparing biological themes among gene clusters. OMICS. mai 2012;16(5):284‑7.

12. Gao S, Mutter S, Casey A, Mäkinen VP. Numero: a statistical framework to define multivariable subgroups in complex population-based datasets. Int J Epidemiol. 1 avr 2019;48(2):369‑74.
